# The Broken Achilles Heel of Behavior Therapy: A Couple of Reflections on the Function Analysis

**DOI:** 10.5334/pb.453

**Published:** 2018-07-26

**Authors:** Paul Eelen, Omer Van den Bergh

**Affiliations:** 1 Faculty of Psychology and Educational Sciences, KU Leuven, BE

**Keywords:** functional analysis

## Abstract

This manuscript is part of a special issue to commemorate professor Paul Eelen, who passed away on August 21, 2016. Paul was a clinically oriented scientist, for whom learning principles (Pavlovian or operant) were more than salivary responses and lever presses. His expertise in learning psychology and his enthusiasm to translate this knowledge to clinical practice inspired many inside and outside academia. Several of his original writings were in the Dutch language. Instead of editing a special issue with contributions of colleagues and friends, we decided to translate a selection of his manuscripts to English to allow wide access to his original insights and opinions. Even though the manuscripts were written more than two decades ago, their content is surprisingly contemporary. This manuscript was originally published in 1992 and discusses functional analysis as the Achilles heel of cognitive-behavioural therapy (CBT). Functional analysis is that part of CBT where insight in learning principles feeds into clinical case conceptualisation. Even though functional analysis was self-evident for first generation behaviour therapists, its importance has been overlooked since long. It is striking to see how modern CBT-approaches again incorporate functional assessment.

First published as: Eelen, P., & Van den Bergh, O. (1992). De gebroken achillespees van de gedragstherapie: enkele bedenkingen bij de functieanalyse. *Psychotherapeutisch Paspoort, 2*, 25–34.^1^

## Introduction

Every behavior therapy training devotes an exceptional amount of attention to the process of learning to set up an adequate function analysis (FA) of the client’s (problem) behavior. Partly due to the influence of Brinkman’s chapter (1978) in *Handboek voor Gedragstherapie*, in which this FA was a central element of the behavior-therapeutic process, this part has continued to have an important place in the training and supervision of behavior therapists, particularly in our (i.e. Dutch) language area. And although consecutive pleas by Burger (1980), Bakker (1987) and Orlemans (1988) did not always have the same understanding of FA, its necessity was never doubted and it was unanimously described as the core element of behavior therapy.

Still, the question remains to what extent the careful setting up of a FA remains present in the practice of current behavior therapists. Sporadic contact and conversations seem to suggest that this important part of the training often has little impact on further therapeutic practice. This suspicion received ample international confirmation in Haynes and O’Brien’s recently published literature overview (1990). The authors consulted 156 case studies published in *Behaviour Therapy* (N = 21), *Journal of Applied Behaviour Analysis* (N = 78), *Behaviour Modification* (N = 31) and *Behaviour Research and Therapy* (N = 26) between 1985 and 1988. In each study it was verified to which extent the reported intervention was justified with a set of data that preceded the intervention. This seems like the minimal requirement in our view to conclude that some form of FA is present. The results of this overview are disappointing. Only 20% of the reported behavior studies meet the chosen criterion. The remaining studies are of the following type: in view of this problem, the following intervention is applied. The cookbook syndrome that behavior therapy has (is) so often been identified with appears to be accurate, even in this set of published case studies.

The problem not only appears in therapeutic practice, however. FA seems to be also largely absent from research on behavior therapy. In 1977 already, Wolpe noted that the composition of groups in outcome studies often demonstrated an ‘unwitting mixing of sheep and goats’ (p. 2). Not much has changed in the meantime, in spite of the new DSM-III-R (1987). Although this important instrument for outcome research included more behaviour criteria for the classification of syndromes than previous editions, it offered more a topographic than a functional description of problem behavior. No matter how important a topographic description may be (and with its development of careful observation techniques, behavior therapy certainly did not come up short in this regard), its exclusive use offers only illusory uniformity when not complemented with a functional description. A trivial example can illustrate this: Bed-wetting can be described in a rather simple topographic fashion. As soon as a FA is completed for this, this phenomenon can vary from one person to the next. It is consequently a hazardous enterprise to lump all bed-wetters together in one category on topographic grounds only and to prescribe a single intervention. Yet precisely this strategy is standard practice in traditional outcome research.

In light of the above, it does not seem an overstatement to say that the FA is being neglected both in practice and in research. Why is this? Why is our Achilles tendon (we borrowed this description from Wolpe, 1977) so flabby or broken? The first part of this contribution makes a first attempt at answering this question. The second part highlights the extent to which a FA forms an essential part of any empirical research and the resulting implications of this fact for (behavior) therapy.

## Function analysis: From corner stone to stumbling block

The FA has in our view been abandoned because of two factors. First, the vocabulary it uses seems outdated, and second, FA almost inevitably results in a déjà vu effect, irrespective of vocabulary. Both factors are explained in more detail below.

From a historical viewpoint, behavior therapy is a late little brother to behaviorism. This new school only gained more or less definite recognition in 1966, when the first association was established under this banner in the United States (*Association for Advancement of Behaviour Therapy: AABT*). This occurred six years after Mowrer synthesized the learning psychology of those days and tried to describe human and animal behavior using the familiar Stimulus-Response (S-R-) jargon of the then dominant neobehaviorist approach in experimental psychology. No matter how magistral this synthesis was, it can be considered as the final death spasm of a school of thought that no longer seemed fruitful. Behaviorism, which had first been voiced in Watson’s (1913) program declaration and subsequently grew into the dominant model in psychology, began to tear apart at its seams at the end of the fifties and quickly became a dead duck. A different research question and an altogether different vocabulary was gradually introduced in psychology under the influence of developments in linguistics, neuropsychology, and information technology. Psychology again became the study of the ‘human mind’, as it originally was.

And then there was behavior therapy in its infancy, claiming to apply findings from general theoretic psychology – and learning psychology in particular – but continuing to use a vocabulary that dated back to the 1950s. Indeed, on to this day, the completion of a FA employs a series of concepts that are directly borrowed from Mowrer’s previously mentioned work. It is therefore not surprising that behavior therapy was (and is) approached with mixed feelings – from annoyance to pity. Annoyance because it continued to rely so heavily on the behaviorism that many gradually came to describe as a dark chapter in psychology’s short history, and which continues to evoke such associations as ‘bestial’, ‘manipulation’, ‘shallowness’, etc., but also pity because this little brother was forced to develop in an era in which the language and thinking of its ‘parents’ seemed obsolete. And this, precisely, is where the first problem in completing a FA lies. Most psychologists (and psychiatrists a fortiori) are no longer familiar with this vocabulary from their basic training because the symbols it uses were directly borrowed from the behaviorist school of thought. Inevitably, the same problems as in behaviourism are run up against, that is, the adequate conceptualisation of language, meaning, cognition, and so on. A very different vocabulary has emerged for precisely these aspects in contemporary experimental psychology, and while behavior therapy has always pretended to be an application of developments in experimental psychology, this appears to be insufficiently reflected in its conceptual framework. It is understandable then that many people somewhat struggle with the behaviorist jargon. This has resulted in the development of a large group of therapists who reject orthodox behaviour therapy and who are not inclined to use the FA’s technical-behaviorist coloured jargon. If a FA is impossible without the use of these old-fashioned symbols, they would rather forego it. They thus willingly sail under a different flag, such as that of “cognitive therapy”, “directive therapy”, etc.

No matter how much we subscribe to the richness of the learning paradigms as an inspiration source for therapy (Eelen, 1988), we do feel somewhat sympathetic to this viewpoint. It is indeed pointless to attach symbols to phenomena that these symbols were not intended for. Describing ‘thoughts’ with COV’s (‘coverants’, as a contraction of ‘covert operation’) in FA does not increase the scientificity of this analysis. Other symbols such as CS, UCS, S^d^ etc. over time received such a limiting meaning that almost all hope was lost that these concepts would acquire a broader meaning. This not only complicates communication with other schools of thought, it also means that the behavior therapists themselves dispose of a language framework that is at best used only during their study programme or training.

Still, we are inclined to think that there has been too much of a blind focus on the used symbolism, and that the completion of a FA that may or may not be expressed with these symbols was essentially disregarded. FA’s extinction because of its jargon is like the proverbial baby that is thrown out with the bathwater.

In addition to these difficulties at the level of symbolism, another reason likely exists that FA fell into disuse in practice. The rich diversity in behavior is reduced to a limited number of basal recurring categories in the FA. This creates a déjà-vu effect after a while and produces a great temptation to implicitly assume that topographically similar behavior can also be placed into the same functional category. The cover of Burger’s book (1980) includes a diagram with four symbols: +S+, +S–, –S+, –S–. These four symbols (Bakker and Eelen extend this to six) supposedly suffice to create a FA for whichever type of behavior, including that of animals of course. Although every scientific discipline aspires to reduce phenomena to a number of basal categories or fundamental laws, this seems less acceptable when the aim is to reduce the richness of a client’s story into such categories and symbols. Everybody implicitly accepts that a patient’s mother is a much more complex stimulus to a patient than a tone is to a dog. If this tone is a discriminative stimulus (S^d^) to the dog to manifest avoidance behavior, it sounds almost ludicrous to assign a similar function to the mother and to represent her with the same symbol. And, still, this ludicrousness is but a semblance. The learning paradigms and related terminology fulfil a model role in the FA: They have an ‘analogon’ status. Much like everybody knows that humans are not computers, yet it is rather easily accepted that ‘pretending as if’ with a computer might be useful to formalise its behavior using the same conceptual framework. The basal FA categories can fulfil a similar heuristic function to acquire insight – a word we do not hesitate to use – into the dynamics that are particular to behavior.

## What is a function analysis?

Every function analysis (FA) seeks to identify the functional relationship between variables. These variables can be situational or behavioural, or refer to personality traits, hormonal situations, etc. The basic formula for every functional analysis is Y = f(X), with Y representing the phenomenon that is to be explained (dependent variable) and X the predictor variable or independent variable. Every functional relationship between two variables expresses that both covariate, but it in fact does not make any statements about the grounds or foundation for this covariance.

A FA is always the end product of a series of prior observations. The basic formula after all assumes that both the X and Y variables are known. The nature of these observations, however, can differ. The observations can be both the result of extensive measurements as well merely based on impressions prompted by the famed ‘clinical eye’. It is evident that the first allows a more precise functional analysis to be formulated than the latter. No matter the nature of the observations, it remains critical that the X and Y variables can be measured and observed independently from each other. If this is not respected, the result is mere tautology. For instance, it is no use to functionally describe a depressive state as determined by a ‘negative self-image’, when the existence of a depressive state was decided via this negative self-image.

The previously mentioned basic formula can become much more complex when the Y variable is first related to different X variables that also receive a different weight. An example of this is Y = f (aX_1_ + bX_2_ + cX_3_), assuming that Y covariates with three X variables each with their own weight (a, b, c).

No matter how complex the FA, it in principle always allows predictions or prognoses with respect to the Y variable to be made. The prediction value is of course determined by the degree to which the X variables have been fully identified. For instance, it is possible that a functional relationship exists between a Y and X variable, but that the covariance is so minimal that it yields little useful knowledge. This promptly brings us to a point of difference between theoretic and applied research. Theoretic research is more interested in demonstrating the *existence* of covariance, while applied research views both the existence and *degree* of covariance as important. It should be noted here that a FA of a phenomenon is seldom or never exhaustive. This means that it is impossible to distil deterministic statements from a FA, and that probabilistic statements can be obtained at best.

## Function analysis and therapy

A number of restrictions applied to the use of FA in (behavior) therapy are added to these general descriptions.

A first restriction overlaps with the already mentioned criterion of *covariance degree*. Because a change in the Y variable is ultimately the aim, it is rather obvious that one will search for X variables that seem to be fairly convincingly connected to the Y variable. It is fairly easy to statistically determine the variance explained by a specific independent variable in experimental nomothetic research. The degree to which the effect of the independent variable can be replicated can moreover point in the same direction. Although similar statistical techniques exist for (experimental) ideographic research, these are rarely used in practice. At the very least, however, one should attempt to verify the importance of the X variable(s) by aspiring to some kind of ideographic replicability.

Because therapy is aimed at change and not solely aimed at predicting the Y variable, a second restriction is that the therapeutic context ideally seeks *causal* relations between Y and X. A causal relationship after all implies that a change in the X variable(s) represents a sufficient (albeit not necessary) condition to achieve a change in the Y variable.

Finally, a FA is only useful to the extent that the X variables are *controllable* and thus capable of being changed or manipulated. X variables that are important and that have a causal relation to the Y variable cannot always be controlled, however. For instance, it is possible that a certain type of problem behavior (Y) is a function of serious past trauma (X). Such an X variable is of course relevant to a causal analysis, but it does not in itself offer an avenue for change. The therapist will therefore also try to find other X variables that also influence the problem behavior and that he/she or the patient has a firmer grip on.

In light of these restrictions, it is not surprising that during the setting up of a FA, the therapist will preferentially seek X variables that are situationally determined. This does not mean that the experience or thought processes of the concerned patient are disregarded, but such variables are not in themselves verifiable and their causal status is disputable. For instance, when ‘cognitions’ are functionally related to overt behavior, there is considerable danger that correlational rather than causal relations are identified. Ideally, one would continue looking for the situational factors that such cognitions are a function of. It is not surprising from this viewpoint that behavior therapy, more so than any other type of therapy, is concerned with thorough observations of externally visible stimuli and responses. This almost automatically causes one to revert to S-R terminology. In addition, this reflection on the FA makes it clear why behavior therapy prefers to seek connection with an experimental paradigm. Every experiment by definition studies a causal and controllable functional relationship between the independent and dependent variables. Experimental psychology in its totality thus remains the most important foundation for (behavior) therapy precisely because it offers theoretical insights that help understand the connection between X and Y. The use of a wide variety in theories does not have to be a problem in itself, and it certainly does not have to result in the development of different therapeutic schools.

A remarkable ecumenical thought arises by way of conclusion: Is a FA not just the cornerstone of behavior therapy but of therapy altogether? Is it even possible to abstain from FA?
